# Lipids and Trehalose Actively Cooperate in Heat Stress Management of *Schizosaccharomyces pombe*

**DOI:** 10.3390/ijms222413272

**Published:** 2021-12-09

**Authors:** Mária Péter, Péter Gudmann, Zoltán Kóta, Zsolt Török, László Vígh, Attila Glatz, Gábor Balogh

**Affiliations:** 1 Biological Research Centre, Institute of Biochemistry, Eötvös Loránd Research Network, 6726 Szeged, Hungary; peter.maria@brc.hu (M.P.); gudmann.peter@brc.hu (P.G.); torok.zsolt@brc.hu (Z.T.); glatz.attila@brc.hu (A.G.); 2 Hungarian Centre of Excellence for Molecular Medicine, Single Cell Omics ACF, 6726 Szeged, Hungary; zoltan.kota@hcemm.eu

**Keywords:** heat stress, mass spectrometry, lipidomics, fission yeast, trehalose, membrane remodeling, signaling lipids, triglyceride synthesis

## Abstract

Homeostatic maintenance of the physicochemical properties of cellular membranes is essential for life. In yeast, trehalose accumulation and lipid remodeling enable rapid adaptation to perturbations, but their crosstalk was not investigated. Here we report about the first in-depth, mass spectrometry-based lipidomic analysis on heat-stressed *Schizosaccharomyces pombe* mutants which are unable to synthesize (*tps1Δ*) or degrade (*ntp1Δ*) trehalose. Our experiments provide data about the role of trehalose as a membrane protectant in heat stress. We show that under conditions of trehalose deficiency, heat stress induced a comprehensive, distinctively high-degree lipidome reshaping in which structural, signaling and storage lipids acted in concert. In the absence of trehalose, membrane lipid remodeling was more pronounced and increased with increasing stress dose. It could be characterized by decreasing unsaturation and increasing acyl chain length, and required de novo synthesis of stearic acid (18:0) and very long-chain fatty acids to serve membrane rigidification. In addition, we detected enhanced and sustained signaling lipid generation to ensure transient cell cycle arrest as well as more intense triglyceride synthesis to accommodate membrane lipid-derived oleic acid (18:1) and newly synthesized but unused fatty acids. We also demonstrate that these changes were able to partially substitute for the missing role of trehalose and conferred measurable stress tolerance to fission yeast cells.

## 1. Introduction

Abrupt temperature fluctuations are able to disturb cellular homeostasis and cause deleterious effects on cellular infrastructure. Cellular membranes are the primary sensors to such insults and they represent the most thermally sensitive macromolecular structures [1,2,3]. Disturbances in membrane homeostasis are linked to a number of human diseases, like diabetes or metabolic syndrome. The homeostatic maintenance of the membrane physicochemical state is therefore of utmost importance. The heat stress response (HSR) is a universal defense mechanism to reestablish homeostasis. In the era of global warming, the understanding of HSR is more necessary than ever before for both health [4] and economic reasons.

The budding yeast *Saccharomyces cerevisiae* (*S. cerevisiae*) and the fission yeast *Schizosaccharomyces pombe* (*S. pombe*) are extensively used model organisms for studying cellular processes. In yeast, HSR can be characterized by a complex response, including induction of hundreds of genes, transient cell cycle arrest, expression of heat shock proteins (Hsp), and ubiquitin-mediated protein degradation [5,6]. In addition, recent advances suggest that eukaryotic cells use highly specific sensor proteins, e.g., Ire1 or Mga2, to monitor bulk membrane properties, like fluidity, bilayer thickness or intrinsic curvature [7]. The response initiated by these sensors should be as fast as possible; therefore, the adaptation to perturbations relies predominantly on lipid and metabolite remodeling. Indeed, HSR features a very fast metabolic response which results in trehalose accumulation, sphingolipid (SL) biosynthesis and glycerophospholipid (GPL) remodeling [6,8,9,10,11].

Trehalose accumulation is one of the most rapid and intensive metabolic responses in yeast HSR. It is a relatively inert and highly stable nonreducing disaccharide which can be found at high concentrations in anhydrobiotic organisms. Its “career” started as a cell stabilizer in the dry state [12], and emerged so rapidly that a myth grew up about trehalose as a universal protectant and chemical chaperone [13]. Indeed, this disaccharide is important for widespread industrial and medical applications [13,14,15,16,17]. In yeast, trehalose is synthesized in a two-step process in which glucose-6-phosphate and UDP-glucose are converted to trehalose-6-phosphate by trehalose-6-phosphate synthase (Tps), then trehalose-6-phosphate is converted to trehalose by trehalose-6-phosphate phosphatase. To be utilized as a carbon source, trehalase converts trehalose into two molecules of glucose. In *S. cerevisiae*, there are two neutral and one acid trehalases (Nth1p, Nth2p and Ath1p), whereas in *S. pombe* only Ntp1 is known. The balance between the activities of synthesizing and degrading enzymes controls the intracellular level of trehalose [18]. In addition, in *S. cerevisiae*, it was clearly shown that not only trehalose synthesis but also trehalose export to the outer side of the plasma membrane is crucial for adaptation of yeast cells to stress conditions because trehalose on both sides of the bilayer is necessary for survival [17,19]. In the budding yeast Agt1p, a stress-inducible high-affinity H+-trehalose symporter was found to be responsible for trehalose transport, but in *S. pombe*, such a transporter has not yet been discovered. Several studies showed that intracellular trehalose levels and the activities of enzymes of trehalose metabolism strongly increased upon heat shock (HS) in both the budding and fission yeasts [14,20,21,22,23]. After preconditioning at moderate temperatures, elevated trehalose levels correlated not only with increased tolerance to HS [21] but also with resistance to freezing and thawing, dehydration, osmostress, and toxic levels of ethanol [24,25]. Based on experiments conducted on *S. cerevisiae*, a mechanism for the action of trehalose was proposed more than 20 years ago revealing that, during HS, trehalose protects cellular proteins against denaturation and subsequent aggregation, but inhibits the solubilization of protein aggregates and the refolding of partially denatured proteins after the stress ceased [18,22,26,27].

Lipids also play an important role in the stress protection of yeasts. As the temperature changes, yeasts rapidly modify the fatty acyl composition of membrane lipids, a phenomenon long-known as the homeoviscous adaptation [28,29]. The rapid adaptation relies on functional changes of lipid remodeling enzymes including phospholipases, acyl transferases and transacylases, as well as enzymes of de novo fatty acid and lipid synthesis and intracellular lipid transport [30,31]. In order to maintain optimal membrane fluidity upon HS, poikilotherms may decrease the level of unsaturation, increase the average acyl chain length, and/or increase the sterol content [11,31,32,33,34,35]. In addition, the stress-induced de novo SL synthesis represents a similarity that yeasts and mammals share in response to HS [36,37,38,39,40]. Sphingoid bases, the first products on the SL biosynthetic pathway, are well-characterized signaling molecules in yeast HSR. Their induction peaks early, and among others, they are required for translation initiation of Hsps or for sustained trehalose accumulation [41]. *N*-acylation of sphingoid bases leads to ceramide (Cer) production, which is known to induce growth suppression and cell cycle arrest in yeast [42].

Nevertheless, a comprehensive study on the kinetics, adaptiveness, flexibility and relative contribution of lipid changes to yeast HS management, as well as their crosstalk with trehalose accumulation, is missing. *S. pombe* is an attractive model organism for unraveling these aspects because, unlike in *S. cerevisiae*, its Tps homologue is completely dispensable for growth on glucose [43]. In this study, we used high-performance shotgun mass spectrometry (MS) to systematically map the lipidomic alterations of wild-type (WT), trehalose-deficient (*tps1Δ*) and trehalase-deficient (*ntp1Δ*) *S. pombe* strains exposed to a moderate HS for 180 min. In addition to trehalose, we followed the kinetics of hundreds of lipid species and lipid-related metabolites, as well as the level of thermotolerance acquired by cells subjected to short (sub)lethal HS after different periods of preconditioning.

## 2. Results

### 2.1. Trehalose Metabolism Mutants

**Figure 1:**
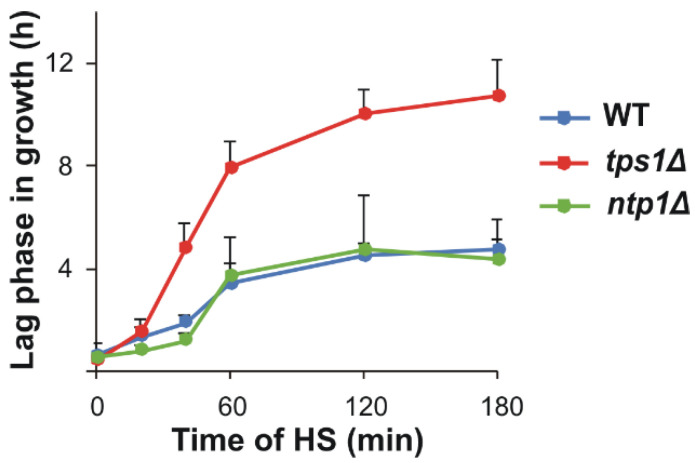
Alteration of lag period due to heat stress (HS). Yeast cells were heat-stressed at 40 °C for 0–180 min. Data are presented as mean ± SD, *n* = 3 (independent experiments); for significance values, see Table S1. WT, wild-type; *tps1Δ*, trehalose-deficient; *ntp1Δ*, trehalase-deficient *S. pombe* strains.

We grew the WT, *tps1Δ* and *ntp1Δ S. pombe* strains for this study on minimal media to mid-log phase at 30 °C to maintain controlled (lipid) metabolic conditions. The cells were heat-stressed at 40 °C for 180 min or, after 60 min of stress, were left to recover at 30 °C for 60 min. Samples were taken at intervals for trehalose quantitation, mass spectrometry-based lipidomic measurements and thermotolerance tests (Figure S1). The mutants did not show any apparent morphological or growth phenotype compared to the WT, and all strains survived the applied heat treatment (Figure 1 and Figure S2). WT and *ntp1Δ* cells displayed a similar lag period in growth following 20–180 min HS at 40 °C, whereas the recovery of *tps1Δ* cells was significantly slower upon > 20 min heat exposure (Figure 1 and Table S1).

### 2.2. Trehalose Accumulation Due to Heat Stress

**Figure 2:**
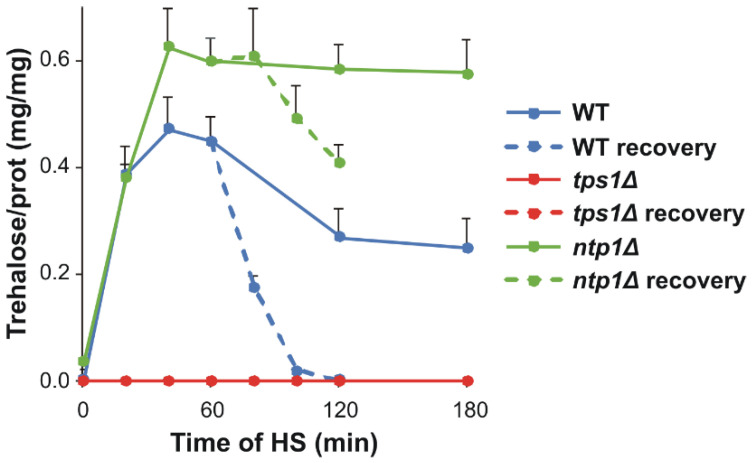
Kinetics of heat-induced trehalose accumulation. Yeast cells were heat-stressed at 40 °C for 0–180 min or, after 60 min of stress, were left to recover at 30 °C for 60 min. Data are presented as mean ± SD, *n* = 3 (independent experiments); for significance values, see Table S2. WT, wild-type; *tps1Δ*, trehalose-deficient; *ntp1Δ*, trehalase-deficient *S. pombe* strains.

In agreement with well-established literature data, trehalose accumulated rapidly and massively during HS in the WT and *ntp1Δ* cells, and reached an induction maximum within 40 min; its level was significantly higher in the trehalase-deficient strain (0.45 and 0.60 trehalose/prot (mg/mg) for WT and *ntp1Δ*, respectively; Figure 2 and Table S2). After 60 min of HS, the disaccharide content started to decrease in the WT, possibly due to the increasing trehalase activity after this time point [25], whereas in the absence of trehalase, trehalose level remained at the plateau. In the recovery phase, after 1 h stress at 40 °C, the trehalose content rapidly returned to the initial, barely detectable level in the WT, but displayed still high, although descending, values in the *ntp1Δ* mutant. We note that the reason for the decline in trehalose during the recovery of trehalase-deficient cells remains to be elucidated in *S. pombe* because enzymes with trehalose transport or alternative hydrolase activities have not yet been identified.

### 2.3. Comparative Shotgun Lipidomics

**Figure 3:**
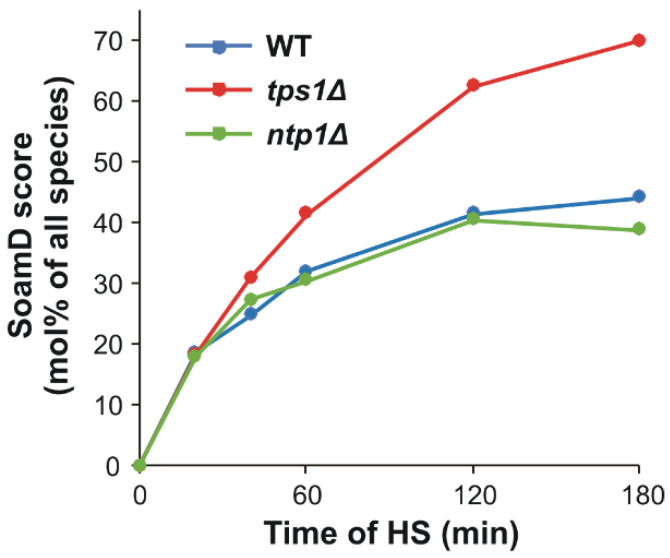
SoamD scores of overall lipidome changes upon heat stress (HS). Yeast cells were heat-stressed at 40 °C for 0–180 min and the sum of absolute mol% differences (SoamD) relative to the untreated control were calculated for each time point based on mean values from *n* = 3 independent experiments as detailed in point 4.6. WT, wild-type; *tps1Δ*, trehalose-deficient; *ntp1Δ*, trehalase-deficient *S. pombe* strains.

To test the role and relative importance of lipid remodeling in HSR, we performed a comparative, mass spectrometry-based shotgun lipidomic analysis on *S. pombe* lipid extracts obtained from stressed and non-stressed WT and mutant cells defective in trehalose metabolism. Our lipidomic platform allowed the broad-range coverage of the yeast lipidome; we identified and quantitated ca. 170 lipid species, including membrane, signaling and storage lipids. The comparison of the molecular species patterns for the different treatment groups revealed hundreds of statistically significant alterations (Tables S3 and S4). To illustrate the extent of heat-induced lipidome rearrangement, we calculated the sum of absolute mol% difference (SoamD) score [44] for each time point to rank the effect of HS on the lipidomes of the studied strains. This calculation showed that the overall lipidome change increased with increasing stress dose and by 180 min of HS reached the value of 70 mol% of all quantitated species in the trehalose-deficient strain compared to 44 and 39 mol% in the WT and *ntp1Δ* cells, respectively (Figure 3).

### 2.4. Trehalose Deficiency Induces More Pronounced Membrane Lipid Remodeling during HS

**Figure 4:**
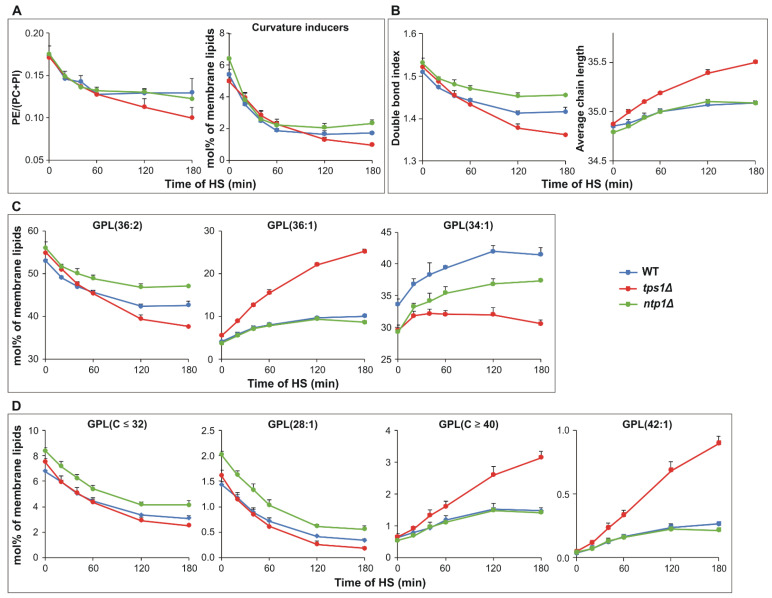
Heat-induced membrane lipid remodeling. Yeast cells were heat-stressed at 40 °C for 0–180 min. (**A**) Changes in PE/(PC+PI) ratio and curvature-inducing lipids comprising lyso GPLs, mono- and dimethyl-PE. (**B**) Alterations in double bond index and average acyl chain length. (**C**) Kinetics of major membrane lipid species GPL(36:2, 18:1/18:1), GPL(36:1, 18:0/18:1) and GPL(34:1, 16:0/18:1). (**D**) Kinetics of medium-chain FA-containing species GPL(C ≤ 32) such as GPL(28:1, 18:1/10:0) and very long-chain FA-containing species GPL(C ≥ 40) such as GPL(42:1, 24:0/18:1). Data are presented as mean ± SD, *n* = 3 (independent experiments); for significance values, see Table S3. WT, wild-type; *tps1Δ*, trehalose-deficient; *ntp1Δ*, trehalase-deficient *S. pombe* strains; GPL, glycerophospholipids; PE, phosphatidylethanolamine; PC, phosphatidylcholine; PI, phosphatidylinositol.

*S. pombe* can use multiple lipid tools to respond to a fluidization stress rapidly and efficiently. First, we focus on the compositional changes of the lipidome (expressed as mol% of membrane lipids), which reports about the membrane physical state. Of the most abundant bilayer-forming GPLs, phosphatidylcholine (PC) showed a tendency to increase, whereas phosphatidylinositol (PI) did not change significantly during the 180 min HS (Table S3). In parallel, we detected a gradual decrease in the relative content of the major non-bilayer forming phosphatidylethanolamine (PE). This resulted in a significant decrease in the PE/(PC+PI) ratio (Figure 4A); this type of membrane stabilization [9] was more pronounced in the absence of trehalose upon longer stress duration (>60 min). In addition, all other curvature-inducing lipids, like mono- and dimethyl-PE, as well as all lyso GPLs, displayed rapid diminishment in all strains (Figure 4A and Table S3). In addition, upon temperature stress, *S. pombe* rapidly reduced the level of membrane unsaturation, characterized by the double bond index, and elevated the average acyl chain length (Figure 4B). The alterations in both parameters were the most remarkable in the absence of trehalose, whereas the modest decrease in double bond index associated with the highest trehalose induction in *ntp1Δ* cells. In addition, we recorded several modifications at the level of lipid species as well (Table S3). While changes in membrane lipid composition usually reached a plateau after 60–120 min of HS in trehalose-containing cells, in trehalose-deficient cells they continued with increasing stress dose (Figure 4 and Table S3). Although the extent of individual responses showed lipid class-dependence (Table S3), the average lipid species profile calculated for the sum of membrane GPLs well reflected the major trends. To reduce the double bond index, *S. pombe* lowered the relative level of the major diunsaturated 36:2 (18:1/18:1) species and, in parallel, increased the level of the monounsaturated 36:1 (18:0/18:1) (Figure 4C). These changes were the most pronounced in *tps1Δ* cells, where 36:2 decreased from 55 to 38 mol%, and 36:1 increased from 6 to 25 mol% during the 180 min of HS. In contrast, the major monounsaturated species 34:1 (16:0/18:1) displayed the least pronounced alteration in the absence of trehalose (Figure 4C). To elevate the average acyl chain length, the cells reduced the relative amount of medium-chain fatty acid (FA)-containing species (sum C ≤ 32), e.g., 28:1 (18:1/10:0), which was accompanied by accumulation in the level of very long-chain FA (VLCFA)-containing species (sum C ≥ 40), like 42:1 (24:0/18:1) (Figure 4D). In the WT and *ntp1Δ* cells, the content of very long-chain components reached a plateau after 60 min, while in *tps1Δ* cells they showed a monotonous rise from 0.7 to 3.2 mol% during 180 min of HS (Figure 4D).

### 2.5. HS-Induced Signaling Lipid Generation Is Enhanced and Sustained in Trehalose-Deficient Cells

**Figure 5:**
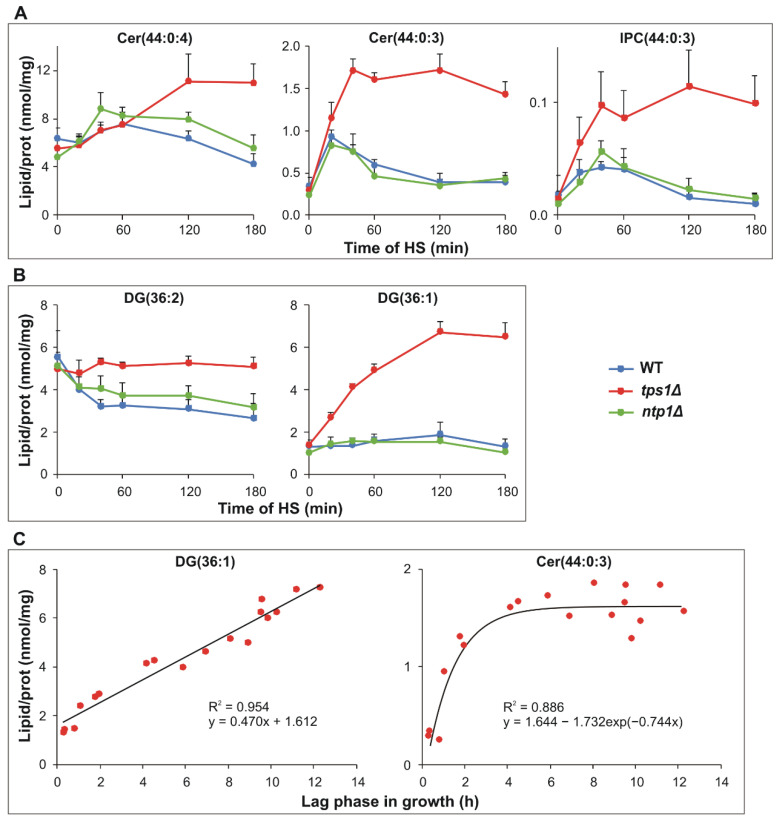
Signaling lipid production in response to heat stress (HS). Yeast cells were heat-stressed at 40 °C for 0–180 min. (**A**) Kinetics of sphingolipid species Cer(44:0:4, t20:0/24:0-OH), Cer(44:0:3, t20:0/24:0) and IPC(44:0:3, t20:0/24:0). (**B**) Kinetics of major diglyceride species DG(36:2) and DG(36:1). (**C**) Relationship between the lag period and signaling lipid levels in *tps1Δ* cells. Black solid lines represent the fitted curves and R^2^ values represent the goodness of fit. Data in panel (**A**) and (**B**) are presented as mean ± SD, *n* = 3 (independent experiments); for significance values, see Table S4. In panel (**C**), individual data are displayed. WT, wild-type; *tps1Δ*, trehalose-deficient; *ntp1Δ*, trehalase-deficient *S. pombe* strains; Cer, ceramide, IPC, inositolphosphoceramide; DG, diglyceride.

Sphingolipid signaling is well-documented in yeast HSR [45]. In agreement with previous data, we detected a rapid and transient accumulation in the major *S. pombe* sphingoid base Sph(20:0:3) (C20 phytosphingosine); its kinetic profile was similar for all strains (Table S4). In contrast, we observed substantially different Cer kinetics in the absence of trehalose (Figure 5A). After 60 min of HS, the major phytoceramide species Cer(44:0:4, t20:0/24:0-OH) showed significant enrichment only in *tps1Δ* cells. Its biosynthetic precursor, Cer(44:0:3, t20:0/24:0), displayed a transient, three-fold elevation at 20 min of HS in the presence of trehalose, whereas in the trehalose-deficient mutant, the level of this species reached more than a 5-fold increase by 40 min of HS, and this high value sustained at later time points. The kinetic profile of inositol phosphoceramide IPC(44:0:3) species was very similar to that observed for its biosynthetic substrate Cer(44:0:3) (Figure 5A). Diglyceride (DG) has various signaling properties as well [46]. The content of the major diunsaturated DG(36:2) showed a significant decrease in the presence of trehalose in the first 60 min of HS; however, in the absence of the sugar, its level did not alter significantly (Figure 5B). In contrast, the monounsaturated DG(36:1) was kept constant in the trehalose-containing strains, while it increased rapidly and reached a plateau in the *tps1Δ* mutant after 120 min of stress. Because these lipids are known to cause cell cycle arrest, we plotted their levels against lag phase length to analyze possible correlations in the absence of trehalose. The obtained plots revealed strong associations for DG(36:1) and Cer(44:0:3), but the type of relationship was different. For DG(36:1), linear regression, whereas for Cer(44:0:3), a non-linear model afforded excellent fit (Figure 5C).

### 2.6. Intensified Triglyceride Synthesis in the Absence of Trehalose upon HS

**Figure 6:**
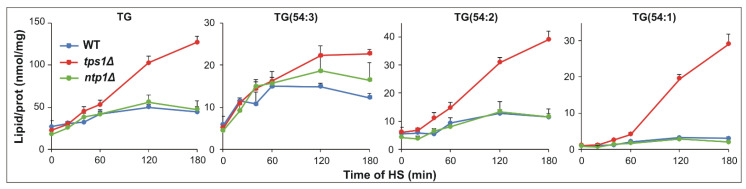
Triglyceride synthesis in heat-stressed *S. pombe* cells. Yeast cells were heat-stressed at 40 °C for 0–180 min. Kinetics of TG as well as major TG species are shown. Data are presented as mean ± SD, *n* = 3 (independent experiments); for significance values, see Table S4. WT, wild-type; *tps1Δ*, trehalose-deficient; *ntp1Δ*, trehalase-deficient *S. pombe* strains; TG, triglyceride.

Triglycerides and steryl esters accumulate in lipid droplets that are dynamic organelles at the center of lipid and energy homeostasis [47]. Previously, we showed that TG synthesis during HS is an active process which facilitates membrane homeostasis by accommodating unsaturated FAs [11]. Here, we observed that TG content reached a plateau after 60 min of HS in the presence of trehalose, whereas it continuously elevated without the disaccharide (Figure 6 and Table S4). At molecular species level, the major trioleoyl species TG(54:3, tri18:1) displayed similar, “plateau-type” kinetics in all strains, with the highest observed maximum in the trehalose-deficient strain. In addition, the abundant saturated 18:0-containing species TG(54:2, 18:0_18:1_18:1) increased much more intensively in *tps1Δ* cells. Similarly, the doubly 18:0-containing TG(54:1, 18:0_18:0_18:1) showed an especially high-fold increase in the trehalose-deficient strain by elevating from 1.1 to 29.1 nmol/prot mg during the 180 min of HS (Figure 6). Ergosteryl ester EE(18:1) did not change in the WT and *ntp1Δ* cells, but displayed significant elevation upon longer stress treatment (>60 min) in *tps1Δ* cells (Table S4), although its extent was much less remarkable than those observed for TG.

### 2.7. De novo Fatty Acid Synthesis and Elongation Have Priority in Heat-Stressed Trehalose-Deficient Cells

**Figure 7:**
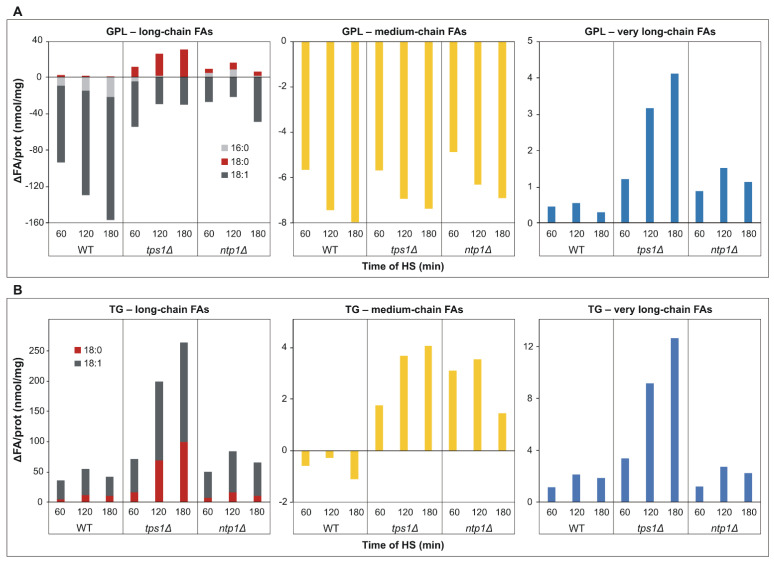
Net lipidome changes at the level of fatty acids (FAs) upon heat stress (HS). Yeast cells were heat-stressed at 40 °C for 0–180 min. Alterations in FA contents were calculated as ΔFA/prot (after HS − before HS) (nmol/mg) values for long-chain, medium-chain (C ≤ 12) and very long-chain (C ≥ 22) FAs (**A**) for GPLs and (**B**) for TG. Averages are shown from *n* = 3 independent experiments. Data were reconstituted based on MS/MS fragmentation results. WT, wild-type; *tps1Δ*, trehalose-deficient; *ntp1Δ*, trehalase-deficient *S. pombe* strains. FA, fatty acid; GPL, glycerophospholipid; TG, triglyceride.

Based on MS/MS fragmentation experiments, we calculated the net changes in the lipidome caused by heat treatment at the level of FAs. This calculation revealed that the most abundant oleic acid (18:1) and medium-chain FAs depleted intensively from membrane GPLs (Figure 7A), and oleic acid depleted from lyso GPLs as well (Figure S3). In parallel, the major FA incorporated into TG was 18:1 (Figure 7B). Remarkably, in *tps1Δ* cells, the net change for the long-chain saturated stearic acid (18:0) and VLCFAs was positive and elevated in time in both GPLs and TG (Figure 7A,B). Furthermore, in the absence of trehalose, the HS-induced DG and SL generation also required significant 18:0 and VLCFA biosynthesis, respectively (Figure S3).

### 2.8. Glycerophosphocholine Induction in the Absence of Trehalose

**Figure 8:**
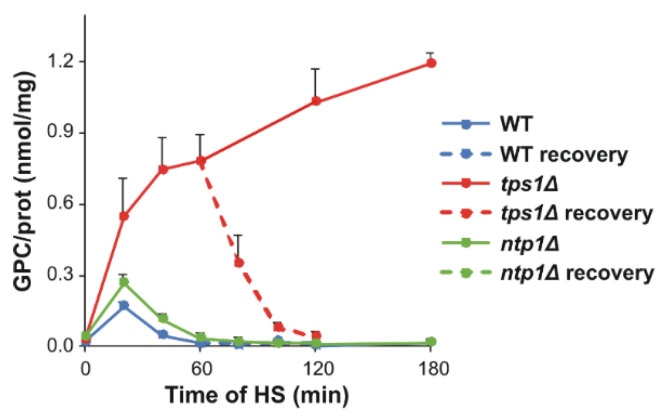
Kinetics of glycerophosphocholine accumulation due to heat stress (HS). Yeast cells were heat-stressed at 40 °C for 0–180 min or, after 60 min of stress, were left to recover at 30 °C for 60 min. Data are presented as mean ± SD, *n* = 3 (independent experiments); for significance values, see Table S2. WT, wild-type; *tps1Δ*, trehalose-deficient; and *ntp1Δ*, trehalase-deficient *S. pombe* strains; GPC, glycerophosphocholine.

Glycerophosphocholine (GPC), a membrane lipid-derived osmoprotectant [48], accumulated slightly and transiently in the presence of trehalose, whereas its concentration gradually increased during the 180 min HS in trehalose-deficient cells (Figure 8 and Table S2). Of note, in the recovery phase, after 60 min HS at 40 °C, GPC level rapidly returned to the initial, very low value in the *tps1Δ* mutant.

### 2.9. Impaired but Sizeable Acquired Thermotolerance in the Absence of Trehalose

Next, we compared the acquisition of thermotolerance for the WT and *tps1Δ* cells. Heat stress at 40 °C was applied as a priming condition, and subsequent short-term exposures to 50 °C (sublethal) or 52 °C (lethal) were used as challenging stresses.

**Figure 9:**
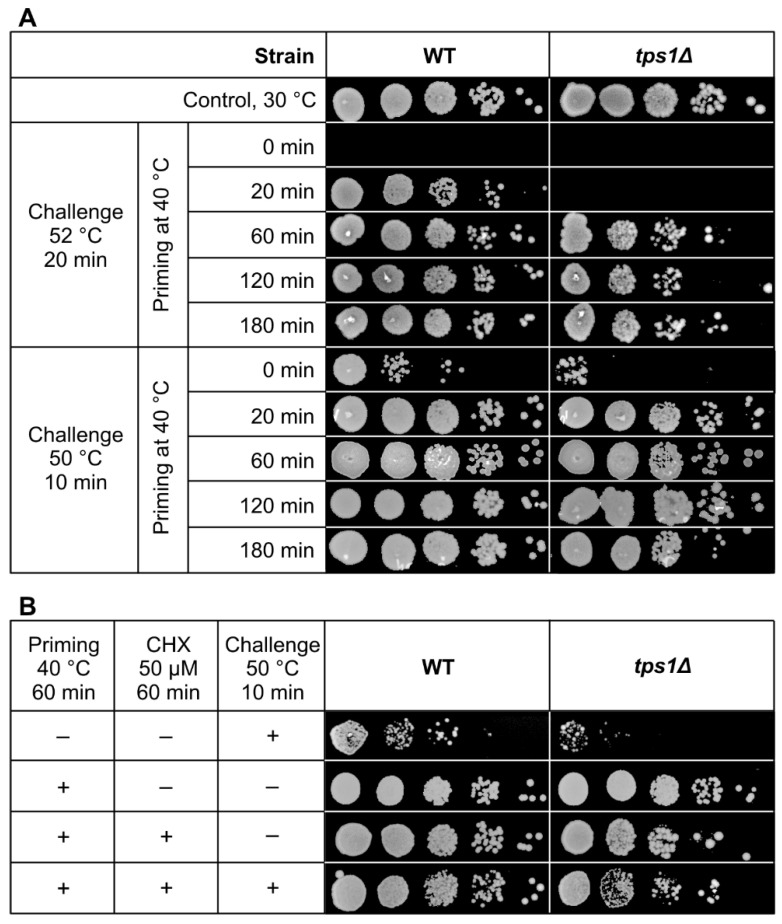
Acquisition of thermotolerance. (**A**) Yeast cells were primed at 40 °C for 0–180 min followed by heat challenge at 52 °C for 20 min or at 50 °C for 10 min. (**B**) Yeast cells were left untreated or primed at 40 °C for 60 min in the absence or presence of 50 μM cycloheximide to inhibit translation followed by exposure to 50 °C for 10 min. Samples were then serially diluted (10×), spotted onto YES plates and incubated at 30 °C for four days. WT, wild-type; *tps1Δ*, trehalose-deficient *S. pombe* strains; CHX, cycloheximide.

Without preconditioning, we detected no resistance against a 20 min challenge at 52 °C for either strain (Figure 9A). Nevertheless, a 20 min priming at 40 °C, where the contribution of Hsp synthesis is subordinate [35,40] but trehalose induction is high in the WT cells (Figure 2), substantially improved the survival of this strain. In contrast, the 20 min priming was not enough to extend the survival of *tps1Δ* cells exposed to 52 °C; therefore, we tested a less harsh condition to be able to identify active defense players in the absence of trehalose. Indeed, when the 20 min priming was followed by a 10 min challenge at 50 °C, both the WT and *tps1Δ* cells acquired virtually complete resistance, whereas the survival was very low in the non-primed controls. Under these priming conditions neither trehalose nor Hsp protection is operational in the *tps1Δ* mutant, but several protective lipid changes, presented above, are significant at this early time point compared with non-primed cells, as illustrated by cluster analysis results (Figure S4).

Upon increasing the length of the priming period, the induction of Hsps becomes more pronounced [35,40]. Indeed, longer preconditioning (60–180 min) conferred practically complete thermotolerance to WT cells exposed to the lethal challenge at 52 °C for 20 min (Figure 9A). The acquired heat resistance was also remarkable in *tps1Δ* cells, although its extent was lower at each time point than those observed for the WT. Of note, survival was also complete in the absence of trehalose when only sublethal challenge was applied at 50 °C for 10 min (Figure 9A). Because in *S. pombe* trehalose accumulation is barely dependent on protein synthesis [21], we proposed that the application of the translation inhibitor cycloheximide would allow dissection of the roles of Hsps, trehalose and lipids at these later time points. However, the presence of cycloheximide remarkably diminished the survival of *tps1Δ* cells when it was applied for longer than 60 min at 40 °C, therefore we used 60 min preconditioning. This experiment revealed again that, in the absence of both trehalose and Hsp induction, *S. pombe* cells acquired measurably higher thermotolerance upon exposure to 50 °C for 10 min than the non-primed controls (Figure 9B). In parallel, we could detect characteristic changes of lipidomic stress protection features, such as the lowering of non-bilayer vs. bilayer-forming lipids (PE/(PC+PI), a decrease in the content of lyso lipids, elevation of very long-chain components or the increase in 36:1 GPL species (Figure S5).

## 3. Discussion

One of the most critical damages that heat causes to cells is the disruption of membrane integrity [49,50]. Here we report about the first in-depth lipidomic analysis on heat-stressed *S. pombe* mutants defective in trehalose metabolism. Most importantly, we showed that besides its well-known role to protect cellular proteins during HS, trehalose protects membranes (membrane lipids) as well.

In *tps1Δ* cells, the HS-induced membrane lipid remodeling was more pronounced and more prolonged than in the presence of the sugar. Among the major protective features, we identified distinguished elevation in the monounsaturated 36:1 (18:0/18:1) species, as well as in VLCFA-containing components. This latter result adds new evidence to the well-documented importance of VLCFA-containing lipids in various cellular functions, e.g., in stabilization of highly curved membranes [34,51,52,53], especially during stress. We should also mention that although the WT and *ntp1Δ* cells displayed similar kinetics for several lipid species, the significantly higher induction level of trehalose in the trehalase-deficient strain associated with clearly less pronounced membrane lipid saturation, i.e., with a lower decrease in the double bond index. Furthermore, unlike in the trehalose-containing strains, the major monounsaturated 34:1 (16:0/18:1) species did not accumulate in the *tps1Δ* mutant, possibly due to the shorter 16:0-FA compared to 18:0-FA in GPL(36:1) components. Consequently, our results show that the extent of membrane lipid remodeling was adjusted to the extent of trehalose induction. Trehalose-deficient cells maximized and optimized these tools to rigidify their membranes in order to reduce the HS-induced hyperfluidization.

We detected remarkable trehalose-dependent differences in the HS-induced signaling lipid generation, too. The most distinctive species were Cer(44:0:3), IPC(44:0:3), and DG(36:1); the changes in these components were more enhanced and sustained in *tps1Δ* cells. Previously, we observed similar alterations in the heat-stressed *S. pombe* mutant unable to synthesize TG [11]. Cer, IPC and DG are known to have versatile signaling properties, all affecting cell growth and viability [42,45,54,55]. We propose that both the defect in membrane protection due to inability to produce trehalose (present study) and the defect in membrane (lipid) remodeling capability due to inability to produce TG [11] contributed to the same physiological outcome, i.e., the transient cell cycle arrest observed upon HS could allow time for cells to recover under suboptimal conditions. Interestingly, we observed different types of relationship between the lag period and the level of DG(36:1) or Cer(44:0:3) in trehalose-deficient cells, which might reflect the activation of different growth arrest pathways. It was also reported for the TG-deficient *S. pombe* mutant that Cer and DG evoked different cell death pathways upon entry into the stationary phase [55]. It should also be mentioned that, unlike in *S. cerevisiae* [18], our data revealed no delay in growth reinitiation of *S. pombe ntp1Δ* cells compared to the WT.

In addition, we documented remarkably higher TG synthesis in the absence of trehalose. The analysis of FA composition revealed sizeable incorporation of de novo synthesized 18:0 and/or VLCFAs into GPLs, SLs, DG and TG. This indicates that in trehalose-deficient cells, de novo FA synthesis (up to 16:0-FA) and elongation had priority to serve the higher need for membrane rigidification and signaling lipid generation. Because the stress dosage could not be foreseen, the unused FAs could be easily stored in TG.

A further remarkable feature that we identified was the generation of GPC, which displayed complementary, trehalose-like kinetics in *tps1Δ* cells, in that it was induced steeply upon HS and degraded quickly after the stress had passed. As the turnover product of PC, GPC is a lipid-derived metabolite, which was shown to be induced by hypersaline stress in *S. cerevisiae* [56], as well as contributing to osmotic adaptation in mammalian renal cells [57]. In addition, radioprotective effect of GPC was also evaluated in a zebrafish embryo model [58]. Consequently, its protective role could be operational in various stresses and in various organisms, a feature that is worth investigating in more detail in the future.

Mild to moderate stress conditions have the ability to improve the survival of cells exposed to a subsequent, otherwise sublethal or lethal insult. Defects in HSR can remarkably deteriorate this benefit and depend on the relative importance and interplay of the individual factors during preconditioning. Hsps, trehalose and lipids are long-known defense components in yeast HSR. Hsps are involved in functions including protein folding, transport, refolding of misfolded proteins, maturation and degradation, cell wall organization, and cell cycle regulation [5]. Indeed, mutants defective in Hsp, trehalose or signaling lipid production or those that cannot adjust properly their membrane lipid composition are all more heat-sensitive than their functional counterparts [11,26,35]. On the other hand, overproduction of individual HSR components could result in enhanced stress resistance. It was shown decades ago that increased trehalose level heightened the stress tolerance of crop plants for agricultural uses [59], Hsp overexpression was associated with elevated survival in mammalian cells [60], or manipulation of membrane lipid composition by catalytic hydrogenation significantly increased the thermostability of thylakoid membranes [61]. Recent research unraveled that unusual asymmetric GPLs impart distinct physical properties to membranes of *Schizosaccharomyces japonicus*, a sister species of *S. pombe*, and confer higher stress resistance to this fission yeast [62,63].

Under HS conditions where both Hsps and trehalose are induced, trehalose protection dominates in the early phase (<60 min), while Hsps protect at the later phase of HS in the WT cells [26,35]. We provided data that lipidome remodeling displayed both early and prolonged alterations; therefore, it overlaps with both trehalose and Hsp inductions. With the exception of very mild HS, trehalose deficiency was associated with lower expression of several Hsps in the budding yeast [64] and adversely affected, under the same stress conditions as applied in the present study, the expression of small Hsps in the fission yeast, too [35]. Not surprisingly, the acquired thermotolerance was impaired in the absence of trehalose in both *S. cerevisiae* [20,26] and *S. pombe* [22]. Compared with the WT, we also observed consistently lower survival for the trehalose-deficient *S. pombe* mutant when it was primed for 60–180 min at 40 °C and then challenged at the lethal temperature of 52 °C. Nevertheless, the resistance acquired by these cells was apparently higher than it could be expected considering the impairment in two major stress defense elements. It is conceivable that the comprehensive lipid changes induced during the preconditioning period could compensate the absence of trehalose (and reduced expression of Hsps) to a considerable extent, and provided sizeable protection against the 52 °C challenge. Remarkably, the 20 min preconditioning, where Hsp expression is subordinate but lipid changes were already significant, could increase the survival of *tps1Δ* cells upon exposure to a sublethal challenge at 50 °C. On the other hand, as opposed to WT cells, the short-term priming could not confer resistance to *tps1Δ* cells against the lethal stress at 52 °C. This observation implies that in the absence of the protein stabilization capacity of the disaccharide, the trehalose-deficient mutant is characterized by a higher global proteome instability upon HS. The higher sensitivity of heat-stressed *tps1Δ* cells to cycloheximide (present study) and the 2-degree downshift in the expression maxima of small Hsps in the trehalose-deficient strain subjected to 40 °C [35] support this suggestion as well. It seems that the enhanced proteome instability sets a threshold above which trehalose is indispensable for survival, especially in the early phase of severe HS.

Moreover, our data support that direct interaction exists between trehalose and lipids. Several biophysical experiments [65,66,67] and simulation studies [68,69] provided evidence for this notion. Most of these reports investigated the dehydrated state of phospholipid liposomes where trehalose stabilizes the bilayer physical state in the absence of water. The substantially higher-degree lipidome rearrangement in the absence of the sugar also implies its direct membrane/lipid phase stabilizing capacity during HS. Hydrogen bonding is a very likely molecular mechanism for trehalose protection [13,68]. The hydroxyls of trehalose might build up several hydrogen bonds with the polar residues of proteins, as well as with the polar headgroup region of membrane lipids. In addition, the drastic stress-induced increase in the intracellular trehalose level causes an increase in cellular osmolarity, which then activates the cell wall integrity pathway [70]. Similarly, direct interaction between Hsps and lipids was also documented [35,71,72,73], suggesting that both trehalose and Hsps protect membranes, and the preservation of membrane integrity has priority during stress. We showed that yeast cells could operate versatile lipid rearrangement tools for the same purpose. It is therefore conceivable that the cooperativity between trehalose, Hsps and lipids ensures a certain extent of redundancy in the maintenance of the proper membrane physicochemical state which, in turn, might confer sufficient protection even with incomplete arsenal of HSR elements.

## 4. Materials and Methods

### 4.1. Materials

Lipidomic standards were from Avanti Polar Lipids (Alabaster, AL, USA). Solvents for extraction and MS analyses were liquid chromatographic grade (Merck, Darmstadt, Germany) and Optima LC-MS grade (Thermo Fisher Scientific, Waltham, MA, USA). All other chemicals were the best available grade purchased from Merck (Darmstadt, Germany).

### 4.2. Yeast Strains and Culture

*S. pombe* strains used in this study are listed in Table S5. BRC1 was used to generate *tps1Δ* and *ntp1Δ* strains according to [35] and [74]. For yeast culture, a small amount of frozen cells was transferred to a YES plate and incubated at 30 °C for 26–30 h. EMM was prepared as in [75], and supplemented with leucine (1.7 mM) and uracil (2 mM). Optical density (OD) was measured at 600 nm on a Biowave Cell Density Meter (CO8000, WPA, Cambridge, UK). Precultures were prepared by inoculating 6 mL of EMM+LU and allowed to grow in a rotary shaker at 30 °C overnight. Cells were then diluted to OD_600_ = 0.5 in 15 mL EMM+LU and allowed to grow for 6–7 h at 30 °C (approx. 2 generation times, OD_600_ = 1.0–1.2). Then, 250–400 μL of the culture was transferred into 100 mL fresh EMM+LU medium and cells were grown at 30 °C (150 rpm; Innova 4230 shaker, New Brunswick Scientific, Edison, NJ, USA). Cells in exponential phase (OD_600_ = 0.5, 3–5 × 10^6^ cells/mL) were used for all experiments.

### 4.3. Heat Stress

*S. pombe* cells were heat-stressed at 40 °C for 180 min or, after 60 min of stress, were left to recover at 30 °C for 60 min. Samples were taken at intervals for trehalose quantitation, mass spectrometry-based lipidomic measurements and thermotolerance tests, as indicated in Figure S1.

### 4.4. Trehalose and Protein Quantitation

For cell lysate preparation, 10^8^ cells were harvested by filtration, resuspended in 300 μL ice-cold water, and disrupted using a Bullet Blender Gold homogenizer (Next Advance, Inc., Averill Park, NY, USA), in the presence of zirconium oxide beads (0.5 mm), at speed 8 for 3 min at 4 °C. For trehalose quantitation, the lysate was boiled for 5 min, centrifuged at 10,000× *g* for 5 min, and a 5 μL aliquot was digested in 45 μL 135 mM citrate buffer (pH 5.6) in the presence of 1.15 mU trehalase (Merck, Darmstadt, Germany) at 37 °C overnight. Glucose content was determined by adding 100 μL of Assay Reagent (GO Assay kit, Merck, Darmstadt, Germany) and incubated at 37 °C for 30 min. Reactions were stopped by the addition of 100 μL 12 N sulfuric acid, and the absorbance was determined by Multiskan EX plate reader (Thermo Fisher Scientific, Waltham, MA, USA) at 560 nm. Trehalose and glucose solutions (25–100 μg/mL) were used as standards. Protein level of the lysates was measured using the Micro BCA^TM^ Protein Assay Kit (Thermo Fisher Scientific, Waltham, MA, USA) according to the manufacturer’s instructions.

### 4.5. Acquisition of Thermotolerance

Cells were subjected to a priming HS at 40 °C for 0–180 min, and then challenged at 50 °C for 10 min (sublethal exposure) or at 52 °C for 20 min (lethal exposure). Samples were then serially diluted (10×) and 10 μL aliquots were spotted onto YES plates and incubated at 30 °C for 4 days. To inhibit translation, cycloheximide was added to the cells 5 min before HS at a concentration of 50 μM.

### 4.6. Mass Spectrometry-Based Lipidomics

A 40-μL portion of the cell homogenate (prepared as in Section 4.4) was immediately subjected to a one-phase methanolic (MeOH) lipid extraction [11] in the presence of 3 μg di20:0-PC as extraction standard. After extraction, the supernatant was stored at −20 °C until MS measurement. Electrospray ionization-MS analyses were performed using an LTQ-Orbitrap Elite instrument (Thermo Fisher Scientific, Bremen, Germany) equipped with a TriVersa NanoMate robotic nanoflow ion source (Advion BioSciences, Ithaca, NY, USA) as described in [11]. For MS measurements, 6 μL of lipid extract were diluted with 144 μL infusion solvent mixture (chloroform:methanol:iso-propanol 1:2:1, by vol.) containing an internal standard mix (Table S6). Next, the mixture was halved, and 5% dimethylformamide (additive for the negative ion mode) or 3 mM ammonium chloride (additive for the positive ion mode) was added to the split sample halves. The lipid classes phosphatidylcholine (PC), lysophosphatidylcholine (LPC), sphingoid base (Sph), diglyceride (DG), triglyceride (TG) and ergosteryl ester (EE) were detected and quantitated using the positive ion mode, while phosphatidylethanolamines (PE, mono- and dimethyl-PE), phosphatidylinositol (PI), phosphatidylserine (PS), their lyso derivatives LPE, LPI, LPS, phosphatidic acid (PA), phosphatidylglycerol (PG), cardiolipin (CL), ceramide (Cer), inositolphosphoceramide (IPC) and mannosyl-inositolphosphoceramide (MIPC) were detected and quantitated using the negative ion mode. We note that free sterols were not assessed in the present study. MS/MS fragmentation experiments were performed as described in [11] and [62]. Lipids were identified by LipidXplorer software [76] by matching the m/z values of their monoisotopic peaks to the corresponding elemental composition constraints. The mass tolerance was set to 2 ppm. Quantitation was made by comparing integrated signal intensities with those of the internal standards after built-in C13 isotopic corrections. Data files generated by LipidXplorer queries were further processed by in-house Excel macros.

Lipid classes and species were annotated according to the classification systems for lipids [77,78]. For glycerolipids, the lipid class (sn-1/sn-2) format specifies the structures of the FA side chains as well as the side chain regiochemistry, e.g., PC(18:0/18:1). In sum formulas, e.g., PC(36:1), the total numbers of carbons followed by double bonds for all chains are indicated. For sphingolipids, first the number of hydroxyl groups in the long chain base (e.g., “t” for the three hydroxyls of phytosphingosine), the number of carbon atoms, and then the number of double bonds are indicated followed by the *N*-acyl chain composition, such as Cer(t20:0/24:0). The sum formula, e.g., Cer(44:0:3), specifies first the total number of carbons in the long chain base and the FA moiety, then the sum of double bonds in the long chain base and the FA moiety, followed by the sum of hydroxyl groups in the long chain base and the FA moiety.

Lipidomic data were expressed as mol% of membrane lipids, where membrane lipids were calculated as the sum of glycerophospho- and sphingolipids, or as absolute quantities expressed as lipid/prot (nmol/mg) values, which were calculated based on the amount of the extraction standard. FA/prot (nmol/mg) values were derived from lipid/prot (nmol/mg) values based on MS/MS fragmentation experiments. PE/(PC + PI) ratio, double bond index and average acyl chain length are dimensionless parameters. Double bond index and average acyl chain length were calculated for fully acylated GPLs as Σ(db x [GPL(*i*)])/Σ[GPL(*i*)] and Σ(C x [GPL(*i*)])/Σ[GPL(*i*)], respectively, where db is the total number of double bonds and C is the total number of carbons in fatty acyls in a given GPL species *i*, and the square bracket indicates mol% of GPLs. SoamD score, sum of absolute mol% differences, was calculated for each strain and for every time point as SoamD(*t*) = Σ(abs([Spec(*i*,*t*)]–[Spec(*i*,*t0*)])), where [Spec(*i*,*t*)] indicates the mol% of lipid species *i* at time point *t*, [Spec(*i*,*t0*)] indicates the mol% of lipid species *i* at time point *t* = 0, and the absolute values of the differences were summarized for all quantitated lipid species.

### 4.7. Statistics

Lag phase, trehalose, GPC and lipidomic results are presented as mean ± SD. Student’s *t*-tests were performed for pairwise multiple comparisons; significance was determined according to Storey and Tibshirani [79] and was accepted for *p* < 0.05 corresponding to a false discovery rate < 0.034. Multivariate statistical analysis of lipidomic datasets was performed using MetaboAnalyst [80].

Length of lag phase was calculated according to [81] and [82] with the Solver add-in in Microsoft Excel. For lag period vs. signal lipid level plots, curve fitting was performed with the Solver add-in in Microsoft Excel; R^2^ values were calculated according to [83].

## 5. Conclusions

It is well-documented that high trehalose level correlates with increased tolerance to various stresses in yeast [17,20,21,25,65,84] and other fungi, as well as in prokaryotes, nematodes and plants [85,86,87]. Because stressors, like high temperature, desiccation, high doses of alcohol, oxidants or selenium supplementation [88], all affect membrane integrity, the disaccharide can be considered as a universal stress-responsive membrane protectant. By using the fission yeast *S. pombe* as a model organism, here we studied the heat-induced crosstalk between trehalose and lipid metabolism for the first time. We showed that in the absence of trehalose, HS induced a comprehensive, distinctively high-degree lipidome reshaping in which structural, signaling and storage lipids acted in concert, and were able to substitute for the protective role of trehalose at a sizeable extent. A recent study revealed that large organellar changes occur during mild HS in the budding yeast [89]. By applying high-content microscopy techniques, studies are also in progress in our laboratory to explore the alterations in (sub)organellar membrane organization and organellar architecture underlying the observed lipidomic changes. In addition, our lipidomic data also suggest that in the absence of the membrane stabilizing capacity of trehalose, the membrane is more disordered. Therefore, membrane property sensors [7] might “feel” the environmental temperature higher than the actual and initiate a more intensive lipidomic response to cope with stress. Unraveling further the role and mechanism of these sensor proteins may open up new ways to manipulate membrane lipid composition in order to increase the stress resistance of organisms. We believe that the new information we provided about the interplay of trehalose and lipid metabolism might further extend the utilization possibilities of improved stress tolerance, a very timely topic in the era of global warming. For example, the simultaneous manipulation of lipid composition and trehalose induction can result in more stress resistant yeast species that could be used to increase the fermentative capacity for bioethanol production, or it could also be beneficial for agricultural uses to heighten the stress tolerance of industrial crops. On the other hand, the accumulation of trehalose can be detrimental when it is related to the stress resistance of pathogenic organisms that infect mammals which do not synthesize the disaccharide. It has already been recognized that the investigation of trehalose synthesis can be utilized in the therapeutic development for infectious diseases [17]; based on our new data, we would suggest the consideration of the lipidome flexibility in such studies.

## Data Availability

All data generated or analyzed during this study are included in this published article and its supplementary information files.

## Supplementary Materials

The following are available online at https://www.mdpi.com/article/10.3390/ijms222413272/s1.
