# Supplementary material for: Lipids and Trehalose Actively Cooperate in Heat Stress Management of Schizosaccharomyces pombe
Source: Int J Mol Sci. 2021 Dec 9;22(24):13272. doi: 10.3390/ijms222413272 (PMC8707580; doi:10.3390/ijms222413272)
Supplement: Supplementary file 1 [file ijms-22-13272-s001.zip › ijms-1479206-supplementary/SupplementaryMaterial_Proof.pdf]

Supplementary Material

Lipids and trehalose actively cooperate in heat stress management of *Schizosaccharomyces pombe*

Mária Péter [1], Péter Gudmann [1], Zoltán Kóta [2], Zsolt Török [1], László Vígh [1,*], Attila Glatz [1] and Gábor Balogh [1,*]

[1] Biological Research Centre, Institute of Biochemistry, Eötvös Loránd Research Network, 6726 Szeged, Hungary; peter.maria@brc.hu (M.P.); gudmann.peter@brc.hu (P.G.); torok.zsolt@brc.hu (Z.T.); vigh.laszlo@brc.hu (L.V.); glatz.attila@brc.hu (A.G.); balogh.gabor@brc.hu (G.B.)

[2] Hungarian Centre of Excellence for Molecular Medicine, Single Cell Omics ACF, 6726 Szeged, Hungary; zoltan.kota@hcemm.eu (Z.K.)

* Correspondence: vigh.laszlo@brc.hu (L.V.); balogh.gabor@brc.hu (G.B.)

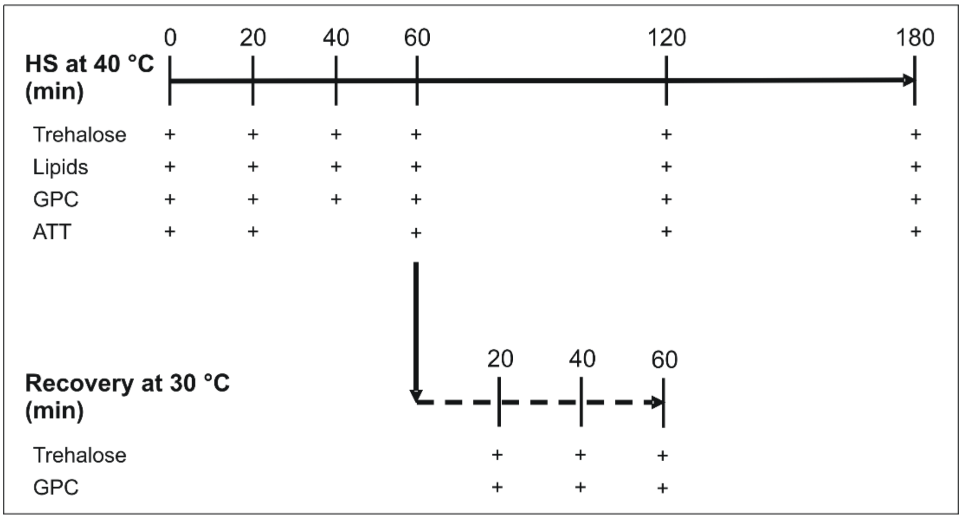

Figure S1. Experimental setup. WT, *tps1Δ* and *ntp1Δ S. pombe* cells were heat-stressed at 40 °C for 0–180 min or, after 60 min of stress, were left to recover at 30 °C for 60 min. Samples were taken at the indicated timepoints for trehalose quantitation, mass spectrometry-based lipidomic measurements, glycerophosphocholine (GPC) determination, and for acquisition of thermotolerance (ATT) as specified.

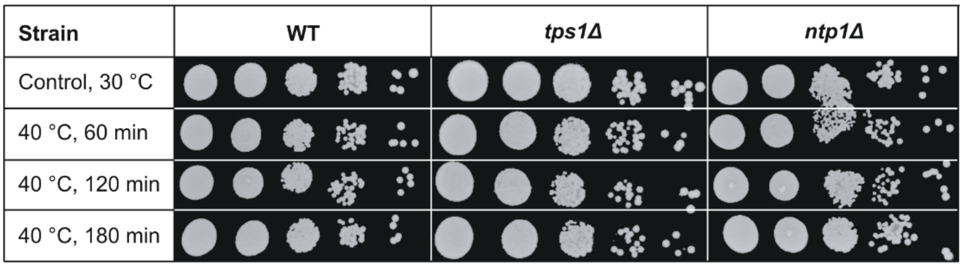

Figure S2. Survival after 0–180 min HS at 40 °C. Cells were exposed to HS, then samples were serially diluted (10×), spotted onto YES plates and incubated at 30 °C for 4 days. WT, wild-type; *tps1Δ*, trehalose-deficient; *ntp1Δ*, trehalase-deficient *S. pombe* strains.

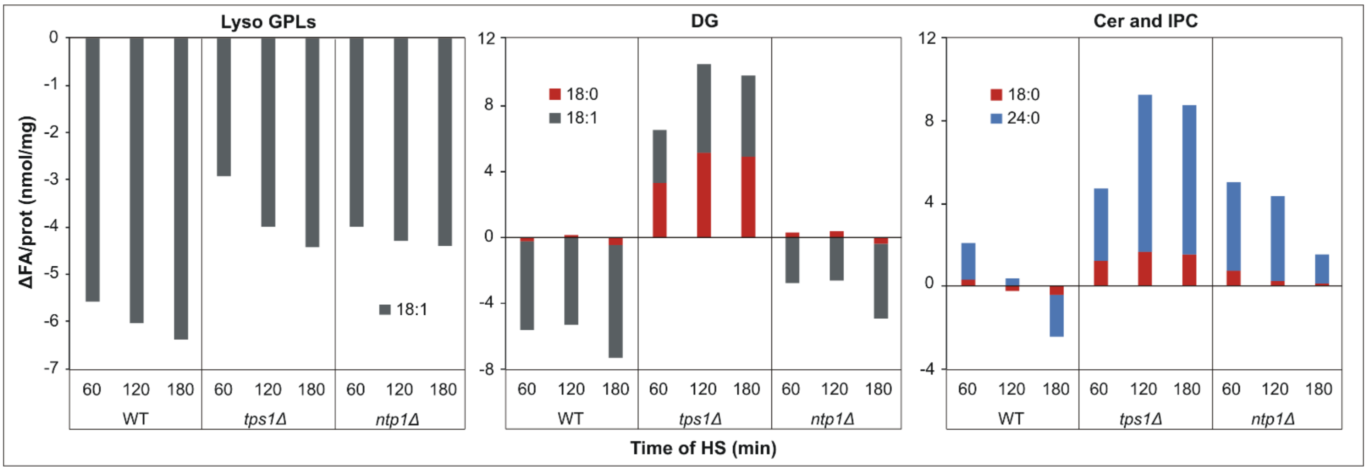

Figure S3. Net lipidome changes at the level of FAs upon heat stress (HS) for lyso GPLs, DG, Cer and IPC. Yeast cells were heat-stressed at 40 °C for 0–180 min. Alterations in FA contents were expressed as ΔFA/prot (after HS − before HS) (nmol/mg) values. Averages are shown from n = 3 independent experiments. Data were reconstituted based on MS/MS fragmentation results. WT, wild-type; *tps1Δ*, trehalose-deficient; *ntp1Δ*, trehalase-deficient *S. pombe* strains. FA, fatty acid; GPL, glycerophospholipid; DG, diglyceride; Cer, ceramide; IPC, inositolphosphoceramide.

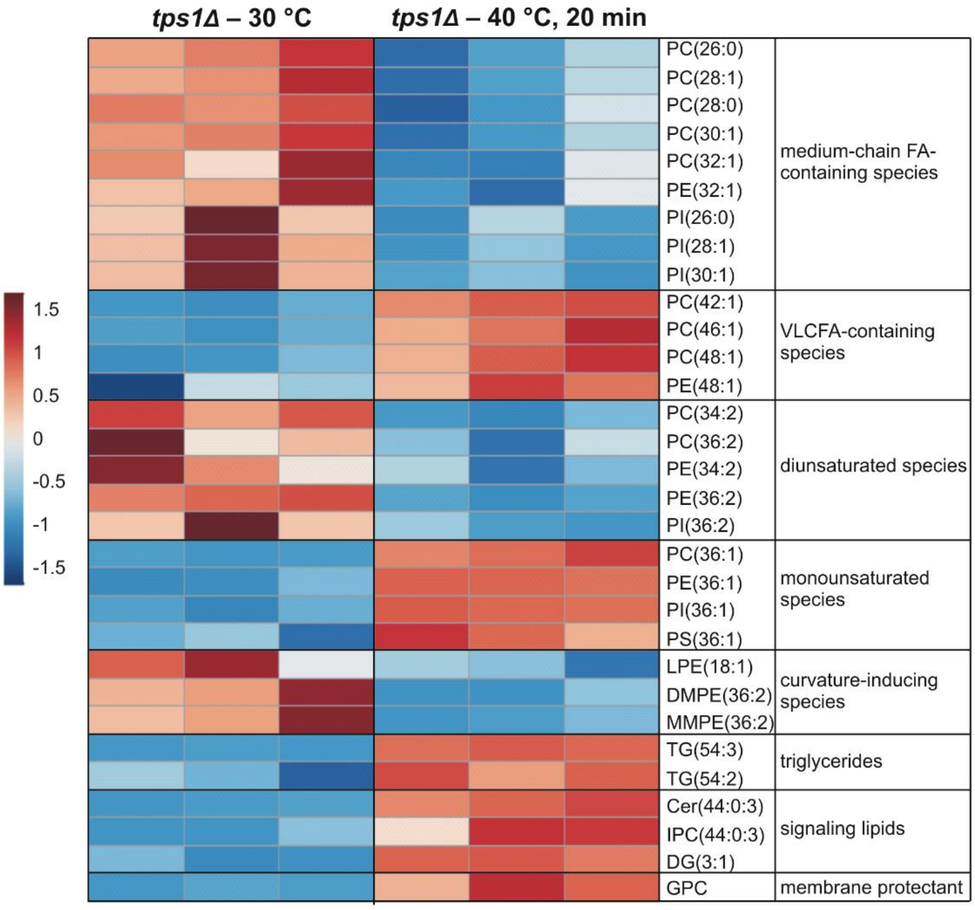

Figure S4. Heatmap representation of hierarchical cluster analysis of lipidomic datasets. *S. pombe tps1Δ* cells were heat-stressed at 40 °C for 20 min or left untreated. Distance measure, Euclidean; clustering algorithm, Ward; heat color code represents normalized values (z-scores).

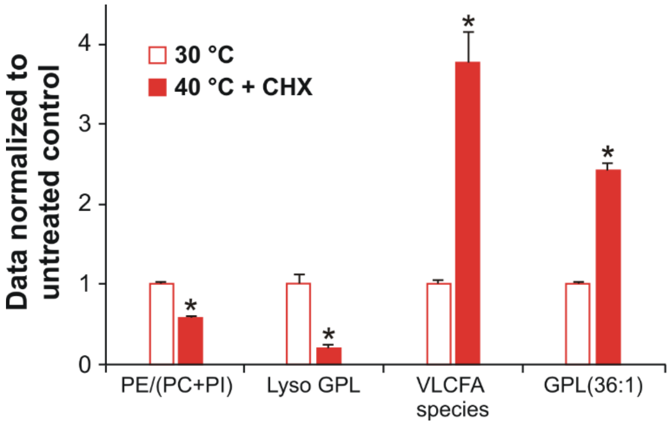

Figure S5. Lipidomic stress protection features in the absence of both trehalose and Hsp production. *S. pombe tps1Δ* cells were heat-stressed at 40 °C for 60 min in the presence of cycloheximide or left untreated. PE/(PC+PI) ratio as well as the mol% of lyso GPLs, VLCFA-containing species, and GPL(36:1) species were normalized to the untreated control. Data are presented as mean ± SD, n = 3 (independent experiments); * denotes $p < 0.001$. PE, phosphatidylethanolamine; PC, phosphatidylcholine; PI, phosphatidylinositol; GPL, glycerophospholipid; VLCFA, very long-chain fatty acid; CHX, cycloheximide; Hsp, heat shock protein.
